# Supplementary material for: Identification of new Dickeya dadantii virulence factors secreted by the type 2 secretion system
Source: PLoS One. 2022 Apr 13;17(4):e0265075. doi: 10.1371/journal.pone.0265075 (PMC9007343; doi:10.1371/journal.pone.0265075)
Supplement: S1 Fig — The sequences of D. dadantii SvfA (Dda3937_01687) and SvfB (Dda3937_00585), D. fanghzongdai SvfC (CVE23_15565), B. cereus WP-193674364.1 and Photorhabdus asymbiotica CAQ86327.1, without their signal sequence, were aligned with Clustal omega. Identical residues are indicated by a star and chemically equivalent residues by a double dot. (DOC) [file pone.0265075.s001.doc]

SvfA -------AQTS----DEPVQTVITALD--SPFVDYPLSAGSEQDVSSSEHKALRAPAPAL 47

SvfC ------------------------------------------------------APAPAL 6

Bacillus ------------------------------------------------------APAPPL 6

SvfB GYSQTINNTDEAGPPIELG---LTAFD-SL-ESNSPLSAARDTS-RASTSGALRAPAPAL 54

Photorhabdus --------QTEQLPKVPAAQKVLIEQSQLLPNVKSPIR-AEERD-LKIEDGSVHAPAPGL 50

**** *

SvfA SSVQVYAVYSSLKGGWQAVPT-NTLSL-SGYAGGTLRIAVLEVGYGGNRIGWLNGGQTS- 104

SvfC SGLRIKVLSGVYGGTWQYAPV-NAVSIGPGYAGGTLQIAVVETGYGGNRIGWINGEQKK- 64

Bacillus TSLNVVKVESQL-GGVEFIGA-NNLSTVKDHGGSYLYIYTNEMGYGRNPIAQMSGQKLKK 64

SvfB SRVTVYAVGSSN-CGWEYMTSIGQLSTTCDHGGAQLRVAVQEIGYGNNPVAWMNGGVLPR 113

Photorhabdus TNMWVYAVGSTN-CGWEYTSNL--FATTCDHGGQQLRAAVLEIGYGYSSFAWMNGGLLPN 107

: : : : . : .: .:.* * . * *** . .. :.*

SvfA --PYQVNPVCVVSGRYTESCPAGSIVSGWMAYFNADNMSSVTFRYQSTSTNFPNRTLSTS 162

SvfC --PDSVKLACLVKGEMTDNCPRGATGAGWIAYFSANYQTSVTFRYQSTSANFPYKTLSTS 122

Bacillus VDS---KMI---------DINGDRTVDGWEYKWDASGQQNGQFKYQNTSTNAPWNTLFTS 112

SvfB SANYQTDGICIVGNQYTFPCPAGYTVVGWMYYYNLDGTDNGQFKFQDTSTNAPRNTLFTQ 173

Photorhabdus SAMYSSKTVCITNGYYTWPCTAGQTVVGYLHEYNLDGNQNGTFRYQNTSTNSPWNTMSVQ 167

. . *: :. . . *::*.**:* * .*: ..

SvfA LNIQ 166

SvfC LTIK 126

Bacillus LNIK 116

SvfB TYIK 177

Photorhabdus INIL 171

*
