# Supplementary material for: Identification of new Dickeya dadantii virulence factors secreted by the type 2 secretion system
Source: PLoS One. 2022 Apr 13;17(4):e0265075. doi: 10.1371/journal.pone.0265075 (PMC9007343; doi:10.1371/journal.pone.0265075)
Supplement: S1 Table — (DOCX) [file pone.0265075.s002.docx]

**Supplementary S1 table: Strains, plasmids and oligonucleotides used in this study**

*Dickeya dadantii* strains

3937 Wild type Laboratory collection

A350 *rafR ganB* (1)

A3838 *kdgR* ::Mu-CmR (2)

A3845 *pecS*::Mu-CmR (3)

A3846 *pecT*::CmR (4)

A3849 *pir*::CmR Laboratory collection

A4237 *gacA*::CmR (5)

A6417 *svfB* ::CmR This work

A6418 *svfA* ::*uidA*-kanR This work

A6434 *kdgR*::Mu-CmR *svfA*::*uidA*-kanR This work

A6435 *pecS*::Mu-CmR *svfA* :*uidA*-kanR This work

A6436 *pecT*::CmR *svfA*::*uidA*-kanR This work

A6436 *pir*::CmR *svfA*::*uidA*-kanR This work

A6437 *gac*A::CmR *svfA*::*uidA*-kanR This work

A6467 *svfB*::*uidA*-kanR This work

A6469 *kdgR*::Mu-CmR *svfB*::*uidA*-kanR This work

A6470 *pecS*::Mu-CmR *svfB*::*uidA*-kanR This work

A6471 *pecT*::CmR *svfB*::*uidA*-kanR This work

A6472 *pir*::CmR *svfB*::*uidA*-kanR This work

A6473 *gacA*::CmR *svfB*::*uidA*-kanR This work

A6522 *svfA*::*uidA*-kanR *svfB* ::CmR This work

A6533 ∆*outD*  laboratory collection

Plasmids

pGEMT AmpR Promega

pBAD33 CmR pBAD (6)

pBBR-MCS3 TetR (7)

pTdB-OD AmpR *outD* (8)

pBBR-svfA pBBR-MCS3 with *svfA* This work

pBBR-svfB pBBR-MCS3 with *svfB* This work

pBAD-outD pBAD33 with *outD* This work

Oligonucleotides

17176H+ CCTCCTGAGATTAGAGAGAG

17176A CGCGCCGGTGTTTTTCTTGCG

17176H- GGTTAGTGATGGTGATGGTGATGCTGAATATTGAGCGACGTGC

17176XbaF GAACAGGATGTGTCCTCTTCTAGACACAAAGCGCTGCGTGCG

17176XbaR CGCACGCAGCGCTTTGTGTCTAGAAGAGGACACATCCTGTTC

15544L2+ CTAAGAATCAGTCAGTTTGCG

15544L2- AGGCAGATAACGCTACTCGCC

15544H+ ATTTATACTCGCCACCGATGC

15544XmaF GCCTTGCGAGCCCCCGCTCCCGGGCTGTCTCGGGTTACCGTG

15544XmaR CACGGTAACCCGAGACAGCCCGGGAGCGGGGGCTCGCAAGGC

15544H- GGTTAGTGATGGTGATGGTGATGTTTAATATAGGTCTGTGTGAAC

14642H+ GGTGAGGAATAATTCTGGCC

14642H- GGTTAGTGATGGTGATGGTGATGAGGCAGTTGTACTTTTCCAG

VirKH+ TGCCGTATGTGATAGTCACG

VirKH- CCTTAGTGATGGTGATGGTGATGTTGCTTGAAGAAGCGGATATC

**References**

1. Hugouvieux-Cotte-Pattat N, Charaoui-Boukerzaza S. Catabolism of Raffinose, Sucrose, and Melibiose in *Erwinia chrysanthemi* 3937. J Bacteriol. 2009 Nov 15;191(22):6960–7.

2. Condemine G, Robert-Baudouy J. Tn *5* insertion in *kdgR* , a regulatory gene of the polygalacturonate pathway in *Erwinia chrysanthemi*. FEMS Microbiology Letters. 1987 Jun;42(1):39–46.

3. Hommais F, Oger-Desfeux C, Van Gijsegem F, Castang S, Ligori S, Expert D, et al. PecS Is a Global Regulator of the Symptomatic Phase in the Phytopathogenic Bacterium *Erwinia chrysanthemi* 3937. J Bacteriol. 2008 Nov 15;190(22):7508–22.

4. Surgey N, Robert-Baudouy J, Condemine G. The *Erwinia chrysanthemi pecT* gene regulates pectinase gene expression. J Bacteriol. 1996 Mar;178(6):1593–9.

5. Lebeau A, Reverchon S, Gaubert S, Kraepiel Y, Simond-Côte E, Nasser W, et al. The GacA global regulator is required for the appropriate expression of *Erwinia chrysanthemi* 3937 pathogenicity genes during plant infection. Environ Microbiol. 2008 Mar;10(3):545–59.

6. Guzman LM, Belin D, Carson MJ, Beckwith J. Tight regulation, modulation, and high-level expression by vectors containing the arabinose PBAD promoter. J Bacteriol. 1995 Jul;177(14):4121–30.

7. Kovach ME, Elzer PH, Steven Hill D, Robertson GT, Farris MA, Roop RM, et al. Four new derivatives of the broad-host-range cloning vector pBBR1MCS, carrying different antibiotic-resistance cassettes. Gene. 1995 Dec;166(1):175–6.

8. Shevchik VE. Specific interaction between OutD, an *Erwinia chrysanthemi* outer membrane protein of the general secretory pathway, and secreted proteins. The EMBO Journal. 1997 Jun 1;16(11):3007–16.
