# Supplementary material for: Identification of new Dickeya dadantii virulence factors secreted by the type 2 secretion system
Source: PLoS One. 2022 Apr 13;17(4):e0265075. doi: 10.1371/journal.pone.0265075 (PMC9007343; doi:10.1371/journal.pone.0265075)
Supplement: S1 Raw images — (PDF) [file pone.0265075.s003.pdf]

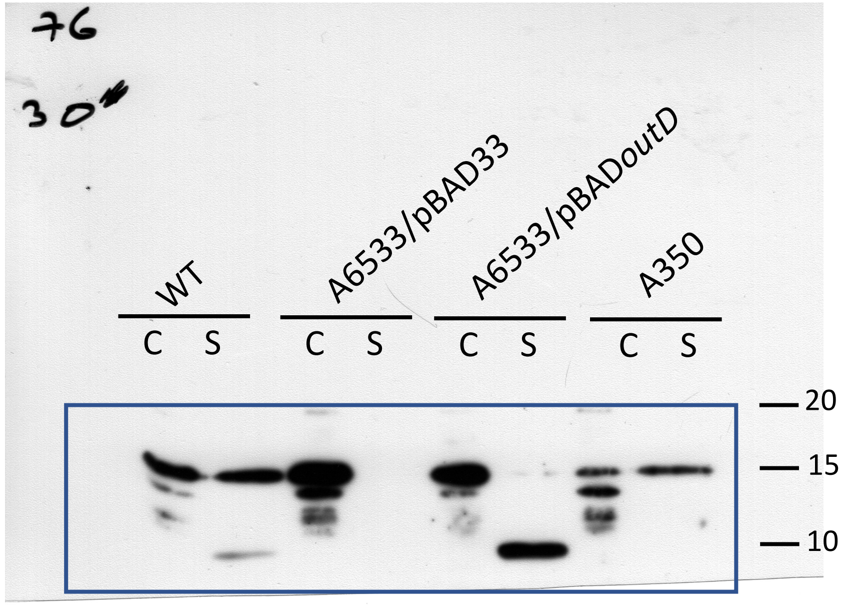

Fig 1 top panel : Western blot for the detection of SvfA with anti-His antibody

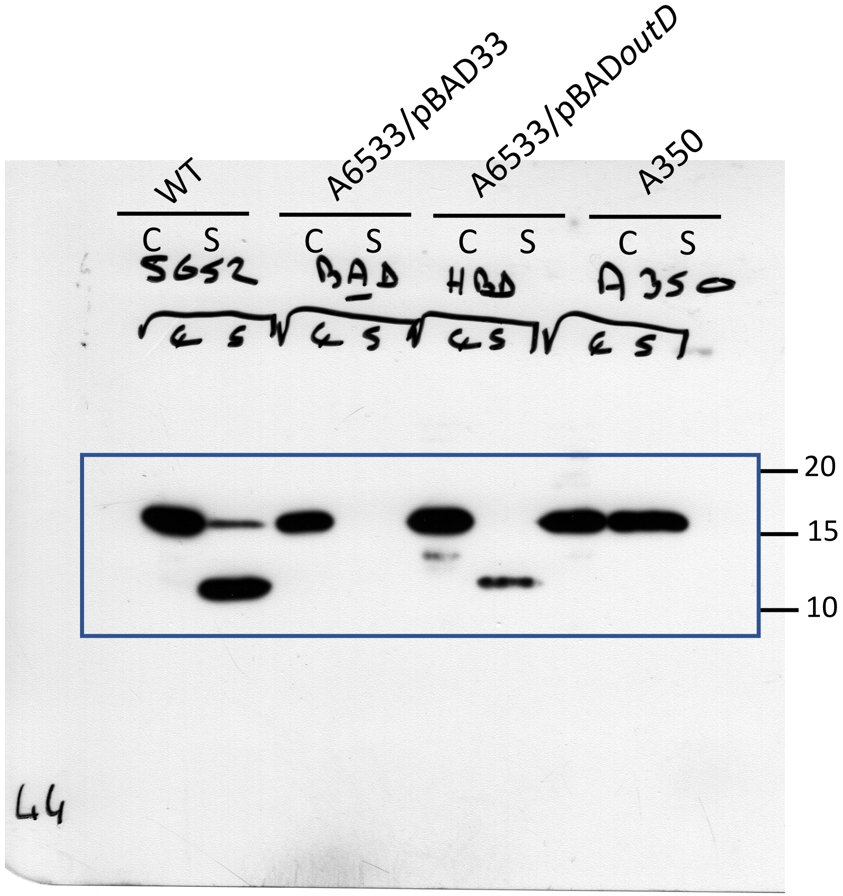

Fig 1 second panel : Western blot for the detection of SvfB with anti-His antibody

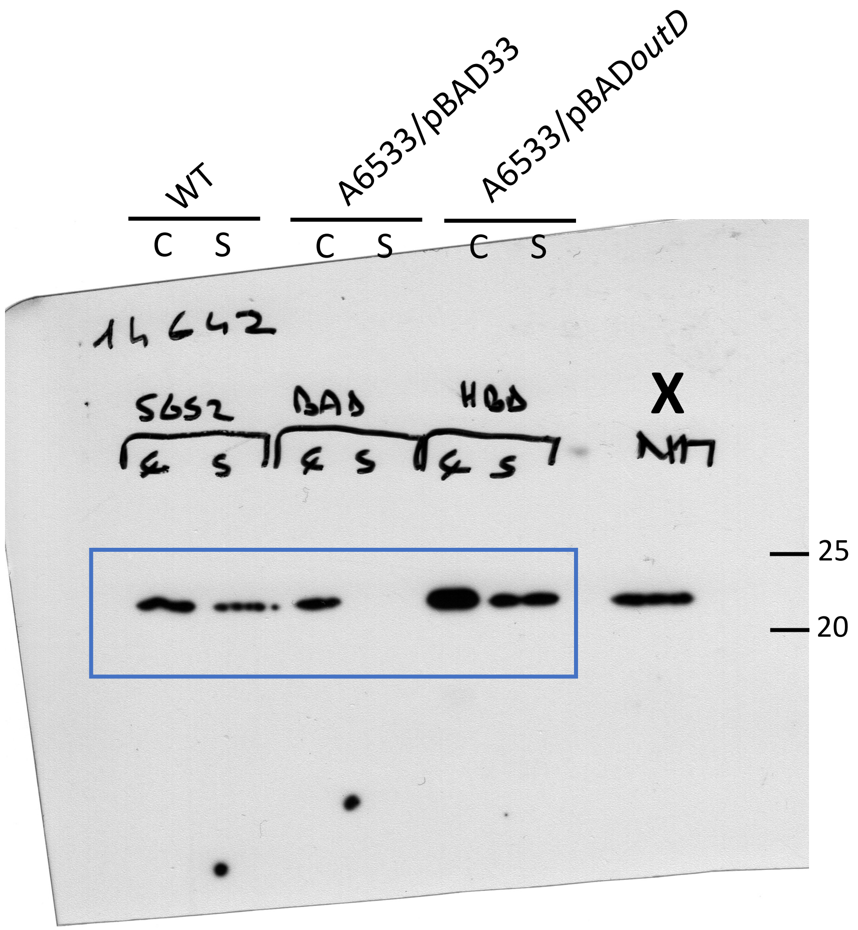

Fig 1 third panel : Western blot for the detection of YolA with anti-His antibody

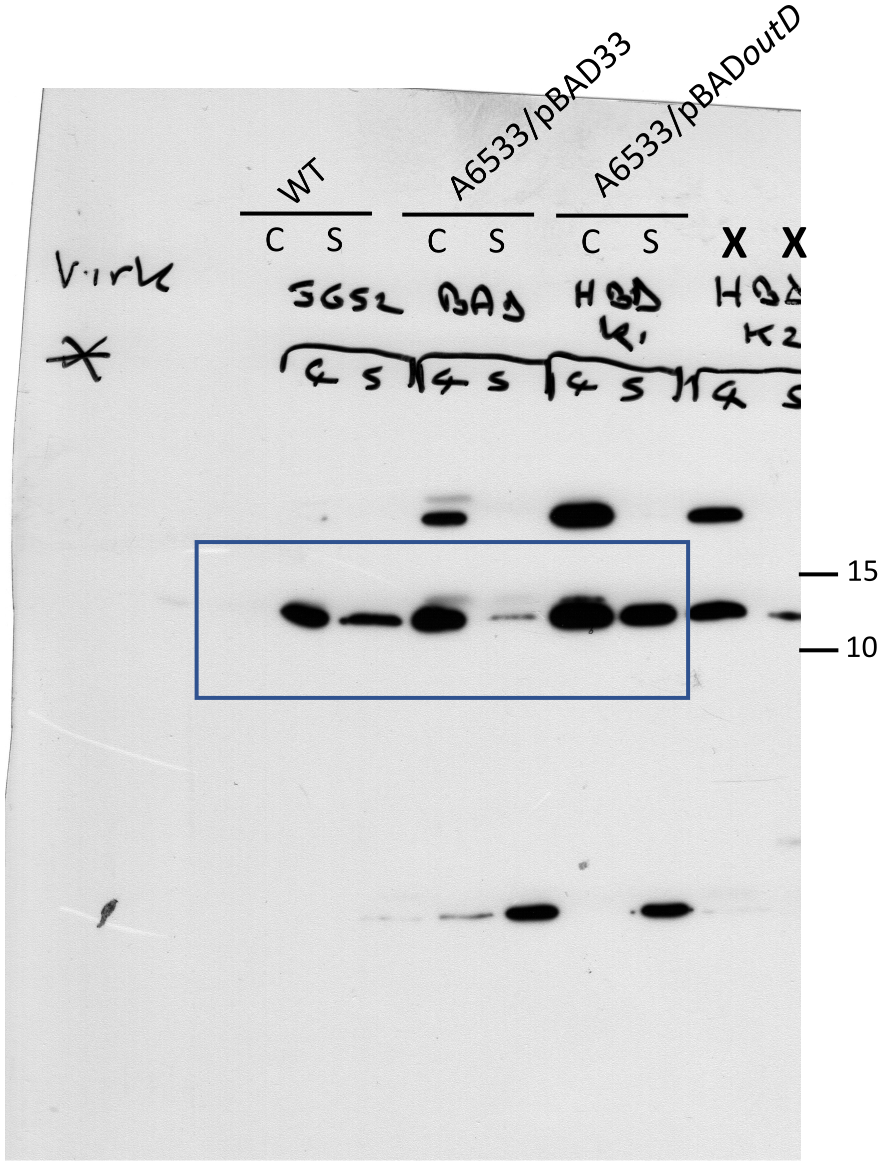

Fig 1 bottom panel : Western blot for the detection of VirK with anti-His antibody
